# The effect of acute moderate-intensity exercise on the serum and fecal metabolomes and the gut microbiota of cross-country endurance athletes

**DOI:** 10.1038/s41598-021-82947-1

**Published:** 2021-02-11

**Authors:** Mariangela Tabone, Carlo Bressa, Jose Angel García-Merino, Diego Moreno-Pérez, Emeline Chu Van, Florence A. Castelli, François Fenaille, Mar Larrosa

**Affiliations:** 1 MAS Microbiota Research Group, Faculty of Biomedical Sciences, Universidad Europea de Madrid, 28670 Villaviciosa de Odón, Madrid Spain; 2 Departamento de Educación, Métodos de Investigación y Evaluación, Universidad Pontificia de Comillas, ICAI-ICADE, 28015 Cantoblanco, Madrid Spain; 3 Université Paris-Saclay, CEA, INRAE, Département Médicaments et Technologies pour la Santé (DMTS), MetaboHUB, 91191 Gif sur Yvette, France

**Keywords:** Microbiology, Physiology

## Abstract

Physical exercise can produce changes in the microbiota, conferring health benefits through mechanisms that are not fully understood. We sought to determine the changes driven by exercise on the gut microbiota and on the serum and fecal metabolome using 16S rRNA gene analysis and untargeted metabolomics. A total of 85 serum and 12 fecal metabolites and six bacterial taxa (*Romboutsia*, *Escherichia coli* TOP498, *Ruminococcaceae* UCG-005, *Blautia*, *Ruminiclostridium 9* and *Clostridium phoceensis*) were modified following a controlled acute exercise session. Among the bacterial taxa, *Ruminiclostridium 9* was the most influenced by fecal and serum metabolites, as revealed by linear multivariate regression analysis. Exercise significantly increased the fecal ammonia content. Functional analysis revealed that alanine, aspartate and glutamate metabolism and the arginine and aminoacyl-tRNA biosynthesis pathways were the most relevant modified pathways in serum, whereas the phenylalanine, tyrosine and tryptophan biosynthesis pathway was the most relevant pathway modified in feces. Correlation analysis between fecal and serum metabolites suggested an exchange of metabolites between both compartments. Thus, the performance of a single exercise bout in cross-country non-professional athletes produces significant changes in the microbiota and in the serum and fecal metabolome, which may have health implications.

## Introduction

Physical exercise has numerous beneficial effects on health^1^. One of the mechanisms by which exercise can exert these effects is through changes to the gut microbiota^2^. While a clear effect of exercise on the gut microbiota has been demonstrated in animal models^3–5^, there is less robust evidence from human studies likely because the performance of physical exercise is associated with different feeding behaviors and with a healthier diet, which is a confounding factor when assessing the response to exercise^6,7^. Exercise is associated with an increase in the diversity of gut microbiota, including an increment in the number of health-promoting bacteria that produce short-chain fatty acids^8,9^. Indeed, acute physical exercise induces a series of metabolic changes both systemically and locally in specific tissues that are characterized by marked shifts in metabolism and oxygen consumption^10^, but its effects on the gut microbiota has not been studied in any great detail.

Advances in metabolomics have provided new opportunities for enriching our understanding of physical exercise-associated factors that stimulate metabolic responses^11,12^. In this line, a recent study by Zhao et al. found that the composition and functionality of the gut microbiota, as measured by fecal metabolomics, changed significantly in amateur runners after endurance exercise (completing a half marathon)^13^. The effect of an acute bout of exercise on the gut microbiota and its interaction with the serum and fecal metabolome has been scarcely investigated. We hypothesized that an acute exercise bout would impact on the serum and fecal metabolome and alter the gut microbiota. Here, using a combinatorial approach, we investigated serum and fecal metabolites and the microbiome in non-professional (cross-country) athletes before and after a session of moderate-intensity exercise to volitional exhaustion using untargeted metabolomics and 16S rRNA sequencing analysis, respectively. We also examined the potential associations between serum and fecal metabolites and the gut microbiota induced by exercise. This study is the first to our knowledge to reveal simultaneous changes in serum/fecal metabolic signatures and gut microbiota after a single acute bout of exercise.

## Results

### Characteristics of the study population, dietary habits and exercise performance

In total, 40 male endurance cross-country runners completed the study. Participants’ characteristics including age, weight, body mass index (BMI), dietary habits and sports performance data obtained in the exercise session are described in Table 1. The exercise session consisted of a treadmill test and running 1 km at maximum speed (see “Material and methods”

**Table 1:** Participant characteristics.

Age (y)	35.79 ± 8.01
Weight (kg)	71.11 ± 8.24
Height (cm)	176.69 ± 6.01
BMI (kg/m^2^)	22.75 ± 2.12
VO_2maxREL_ (mL/kg/min)	58.80 ± 3.24
VT1 (km/h)	13.28 ± 0.95
VT2 (km/h)	15.76 ± 1.01
MAS (km/h)	17.88 ± 1.25
t1km (min)	3.22 ± 0.26
Energy (kcal)	2229.32 ± 1118.74
Carbohydrates (% of energy)	45.41 ± 7.08
Proteins (% of energy)	19.18 ± 3.48
Lipids (% of energy)	35.17 ± 6.85

*VO*_*2maxREL*_ relative maximum oxygen consumption, *MAS* maximal aerobic speed, *VT1* first ventilatory threshold, *VT2* second ventilator threshold, *t1km* time to run 1 km. Values are the mean ± standard deviation.

### Fecal pH and ammonia

Fecal pH and ammonia were determined in samples collected before and after the exercise session. No changes in pH (pre = 7.59 ± 0.48 post = 7.54 ± 0.39; p = 0.337) were detected after the exercise session, but the concentration of fecal ammonia was significantly greater post-exercise (pre = 13.46 ± 7.25 mmol/L; post = 16.08 ± 8.37 mmol/L; p < 0.023).

### Metabolomic serum profile

**Figure 1:**
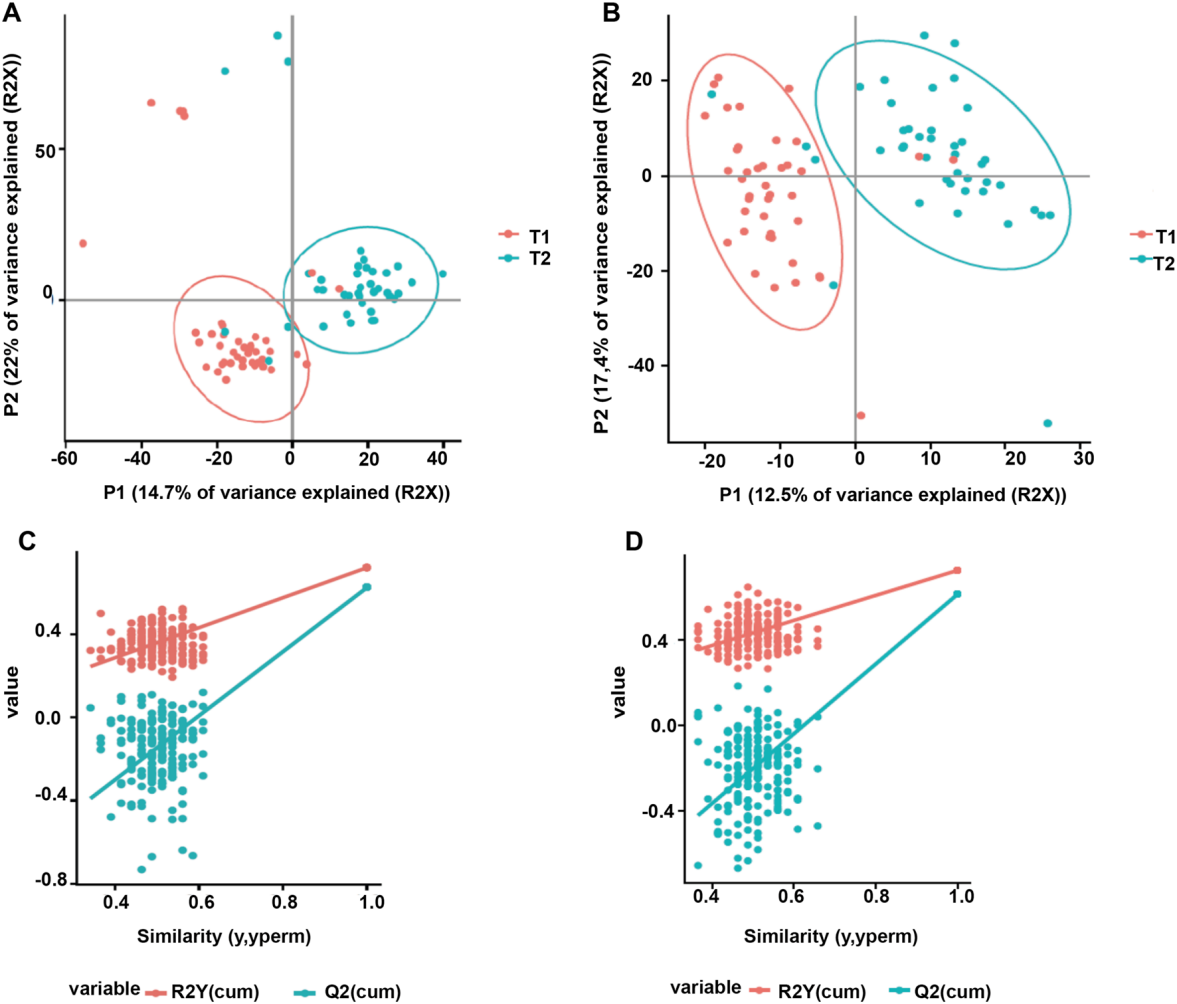
Partial least square discriminant analysis (PLS-DA) of the metabolites in T1 (before exercise) and T2 (after exercise). The results are presented as principal component score plots, with each point in the plot representing an individual sample. (**A**,**B**) PLS-DA score plots obtained from LC-HRMS data in positive mode C18 and negative mode HILIC. (**C**,**D**) Statistical validation of the PLS-DA model (**A**,**B**), showing R2Y (pink dots) and the Q2 (light-blue dots) values from the permutated analysis (bottom left) lower than the corresponding original values (top right).

**Figure 2:**
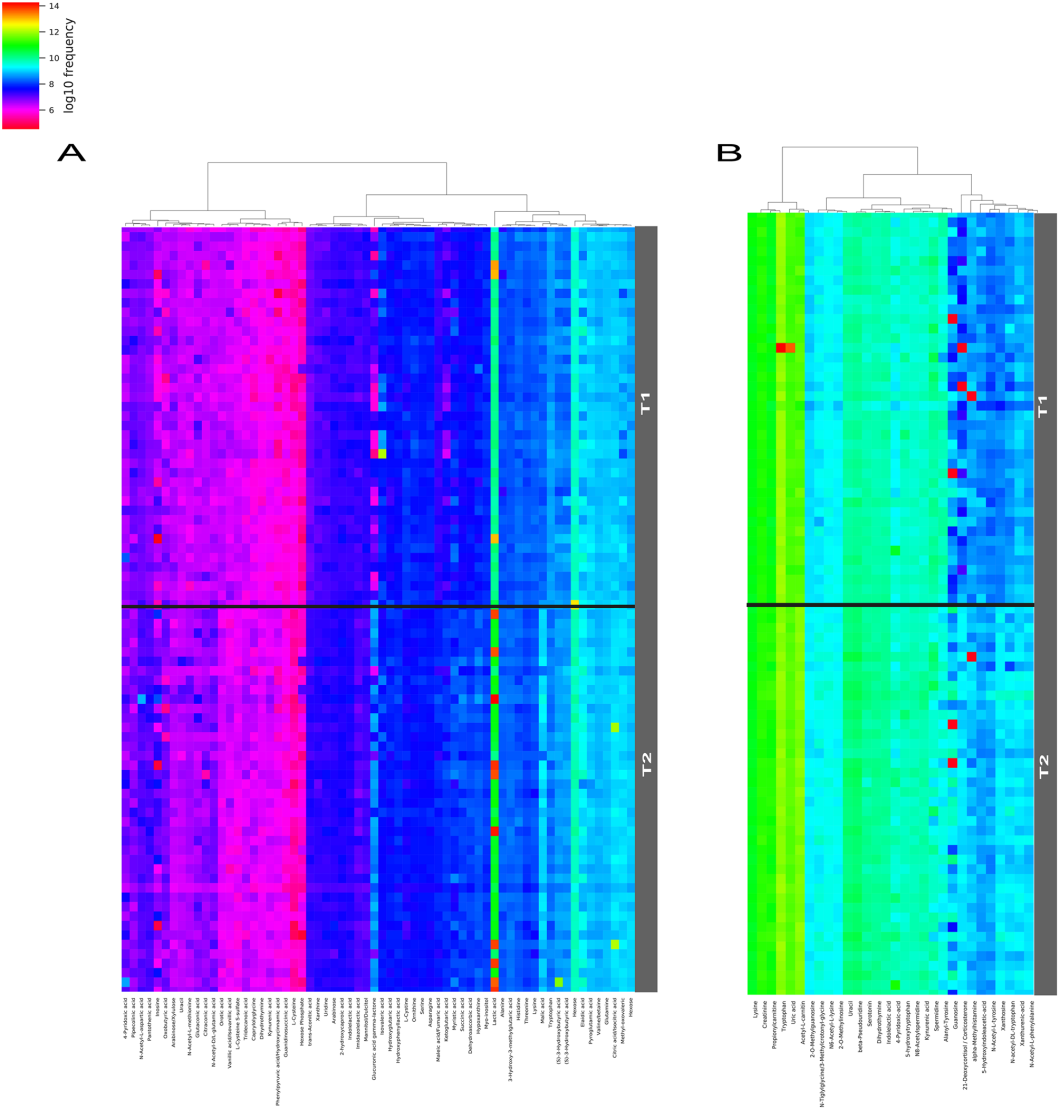
Hierarchical clustering heatmaps representing annotated serum metabolites significantly (p < 0.05) different between T1 and T2. (**A**) Metabolites obtained from HILIC(−) LC-HRMS analysis. (**B**) Metabolites obtained from C18(+) LC-HRMS analysis.

**Figure 3:**
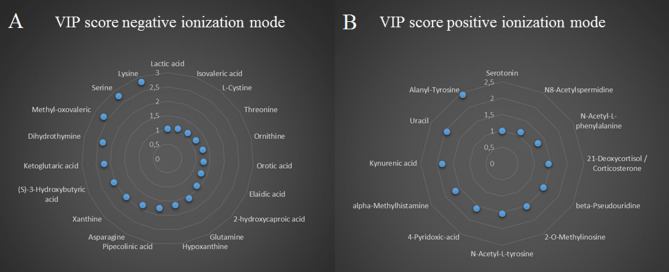
Variable important in projection (VIP) score from PLS-DA of serum metabolites. (**A**) VIP score from serum metabolites obtained in positive ionization mode. (**B**) VIP score of serum metabolites obtained in negative ionization mode.

**Figure 4:**
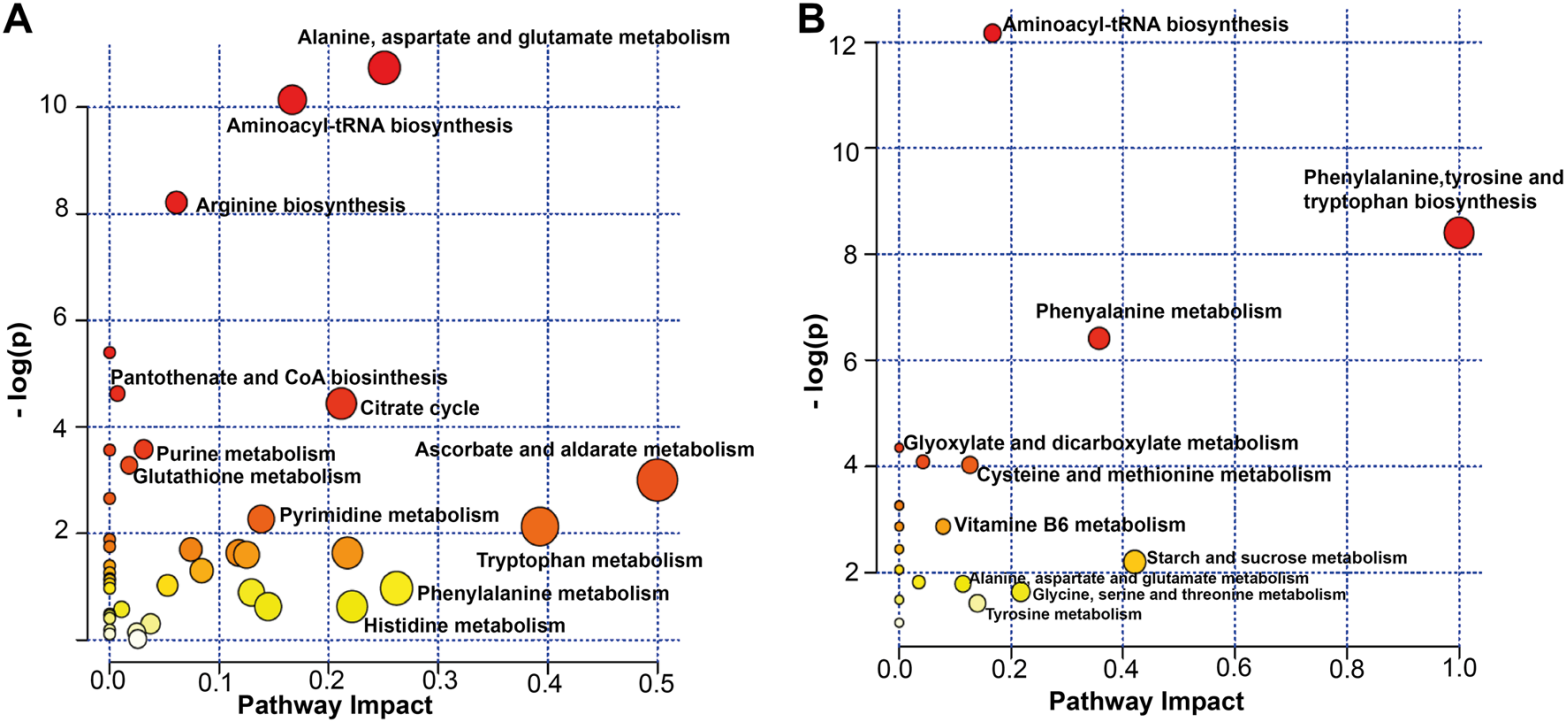
Summary of metabolic pathway enrichment analysis performed in MetaboAnalyst 4.0 using a panel of 85 metabolites found to be significantly altered by a session of acute exercise. All the matched pathways are displayed as circles. The node color is based on its p-value and the node radius is determined based on its pathway impact values. (**A**) Serum pathways modified by a session of acute exercise. (**B**) Fecal pathways modified by a session of acute exercise.

Metabolomics analyses were performed using liquid chromatography coupled to high-resolution mass spectrometry (LC-HRMS) with a combination of two complementary chromatographic methods: reversed-phase chromatography (C18 chromatographic column [C18(+)] and Hydrophilic Interaction Liquid Chromatography [HILIC(−)], for the analysis of hydrophobic and polar metabolites in the positive and the negative ionization modes, respectively). Using an untargeted approach, we detected a total of 3195 and 1600 metabolite features using C18(+) and HILIC(−) conditions, respectively. Among those metabolite features, 101 from the C18(+) and 159 from the HILIC(−) analysis accurately matched the mass and retention time of metabolites included in our chemical database (30 of which were common in both analyses). Differences between the two sampling times were investigated further using supervised partial least square discriminant analysis (PLS-DA). As shown in Fig. 1A,B, athletes’ samples before a session of acute exercise (T1) could be distinguished from those after a session of acute exercise (T2). The cross-validation parameters R2Y and Q2 indicated the variance and the predictive ability of the model. Permutation tests (200 times) were conducted to assess the robustness of the PLS-DA model when using a small sample size (Fig. 1C,D). Whole serum metabolomic profiles from both the negative and positive ionization modes following hierarchical clustering analysis are shown in Fig. 2A,B. Discriminant annotated metabolites between groups were ranked according to their variable importance in the projection (VIP) score, yielding a total of 31 features with a VIP score of > 1, including 13 amino acids, 4 lipids, 5 organic acids, 3 aromatic heterocyclic compounds, 1 aliphatic heterocycle compound, 3 aromatic heteropolycyclic compounds, and 2 nucleosides (Fig. 3A,B). Complementary to the panel of metabolites identified from the multivariate model, univariate analysis was applied using pairwise comparisons (T1 vs T2) of individual metabolites (Wilcoxon p-values with Benjamini–Hochberg correction). Under these conditions, up to 85 annotated metabolites were significantly different and were subjected to additional tandem mass spectrometry (MS/MS) experiments for identity confirmation (Supplementary Table S1). The 85 metabolites were then analyzed by MetaboAnalyst (with the KEGG database^14^) to identify potential discriminately perturbed metabolic pathways before and after the session of acute exercise (T1 vs T2). Results showed that alanine, aspartate and glutamate metabolism, aminoacyl-tRNA biosynthesis and arginine biosynthesis were the three most significantly modified pathways (Fig. 4A, Supplementary Table S3).

### Metabolomic fecal profile

A similar experimental set-up was used for the fecal analysis. A total of 10,866 and 6795 metabolite features were detected under C18(+) and HILIC(−) LC-HRMS conditions, respectively. Firstly, lists of 176 and 335 metabolite features were obtained following annotation with our chemical database and using the C18(+) and HILIC(−) datasets, respectively (88 of which were common). The score plot of the principal component analysis (PCA) and PLS-DA did not reveal any distinction, showing that the two groups did not cluster separately (data not shown). However, 50 annotated metabolites were significantly different when comparing T1 and T2 groups using univariate statistical analysis, and were further confirmed by MS/MS analysis (Supplementary Table S2). Further correction of the p-values for multiple hypotheses using the Benjamini–Hochberg method narrowed down this list to 12 relevant metabolites (Table 2). Additional information for each metabolite is shown in the supplementary material (Supplementary Fig. S1). Metabolic pathway analysis showed that the top 3 affected metabolic pathways after the acute exercise session were the aminoacyl-tRNA biosynthesis, phenylalanine, tyrosine and tryptophan biosynthesis, and the phenylalanine pathway (Fig. 4B, Supplementary Table S4).

**Table 2:** Annotated and top significant fecal metabolites.

LC-HRMS conditions	Metabolite	p-corrected value	T1/T2
C18(+)	Tryptophan	0.0212	0.58
C18(+)	Methionine	0.0132	0.55
C18(+)	*S*-ethyl-l-cysteine	0.0143	0.57
C18(+)	Phenol	0.0145	0.63
C18(+)	4-Hydroxybenzyl-alcohol	0.0234	0.64
HILIC(−)	l/d-Glutamine	0.0001	0.53
HILIC(−)	Serine	0.0212	0.60
HILIC(−)	Phenylalanine	0.0218	0.55
HILIC(−)	Tyrosine	0.0224	0.60
HILIC(−)	Hexose alcohols	0.0224	0.57
HILIC(−)	Pyridoxamine	0.0426	1.66
HILIC(−)	Cinnamic acid	0.0433	0.53

### Changes originating in gut microbiota

Analysis of the gut microbiota before and after the acute exercise session revealed no difference in the composition of the bacterial community, and no changes were observed in any of the determined parameters of β-diversity (Bray–Curtis distance, Jaccard index and weighted and unweighted Unifrac; data not shown). Regarding α-diversity, no changes were observed in any of the parameters analyzed (observed operational taxonomic units, evenness, Shannon index and Faith's index) (Supplementary Fig. S2). To evaluate changes in bacterial taxa, we use an exploratory analysis of the bacterial taxa volatility—an approach that uses machine-learning regressors to establish the important bacterial taxa that predict the T2. The accuracy obtained for our model was significant (Mean squared error = 0.3194; R2 = 0.392; p = 0.0071). The bacterial taxa and those with the higher cumulative average change between T1 and T2 identified by the volatility plot were used to test whether the relative abundances of these features were impacted by exercise using a linear mixed effects analysis, in which time was included as a forced predictor (fixed effect) and subject identifier as a random effect. Six bacterial taxa were identified as significantly differentially abundant between T1 and T2 (Table 3). The exercise bout increased the abundance of *Romboutsia* genus, *Ruminococcocaceae* UCG-005, *E. coli* TOP498 and *Blautia* genus, and decreased the abundance of *Ruminiclostridium 9* and *Clostridium phoceensis*.

**Table 3:** Linear mixed-effects model results for bacterial taxa abundances modified by an acute session of physical exercise.

Bacterial taxa	Estimate	SE	Z-score	P-value	Identity Confidence
*D_5__Romboutsia;D_6__uncultured bacterium*	0.001	0.00	2.390	0.017	0.98558
*D_5__Escherichia-Shigella;D_6__Escherichia coli* TOP498	0.001	0.001	2.534	0.011	0.99624
*D_5__Blautia;D_6__human gut metagenome*	0.000	0.000	3.107	0.002	0.96172
*D_5__Ruminococcaceae UCG-005;D_6__uncultured organism*	0.001	0.000	2.063	0.039	0.99940
*D_5__Ruminiclostridium9;D_6__uncultured Clostridia bacterium*	− 0.000	0.000	− 2.607	0.009	0.99772
*D_5__uncultured;D_6__Clostridium phoceensis*	− 0.001	0.000	− 2.413	0.016	0.99705

Parameter estimate (coefficient), standard error, Z-score, and P-value for each bacterial taxa. A positive coefficient indicates a higher relative abundance in T2.

### Microbiota and metabolomic associations

**Figure 5:**
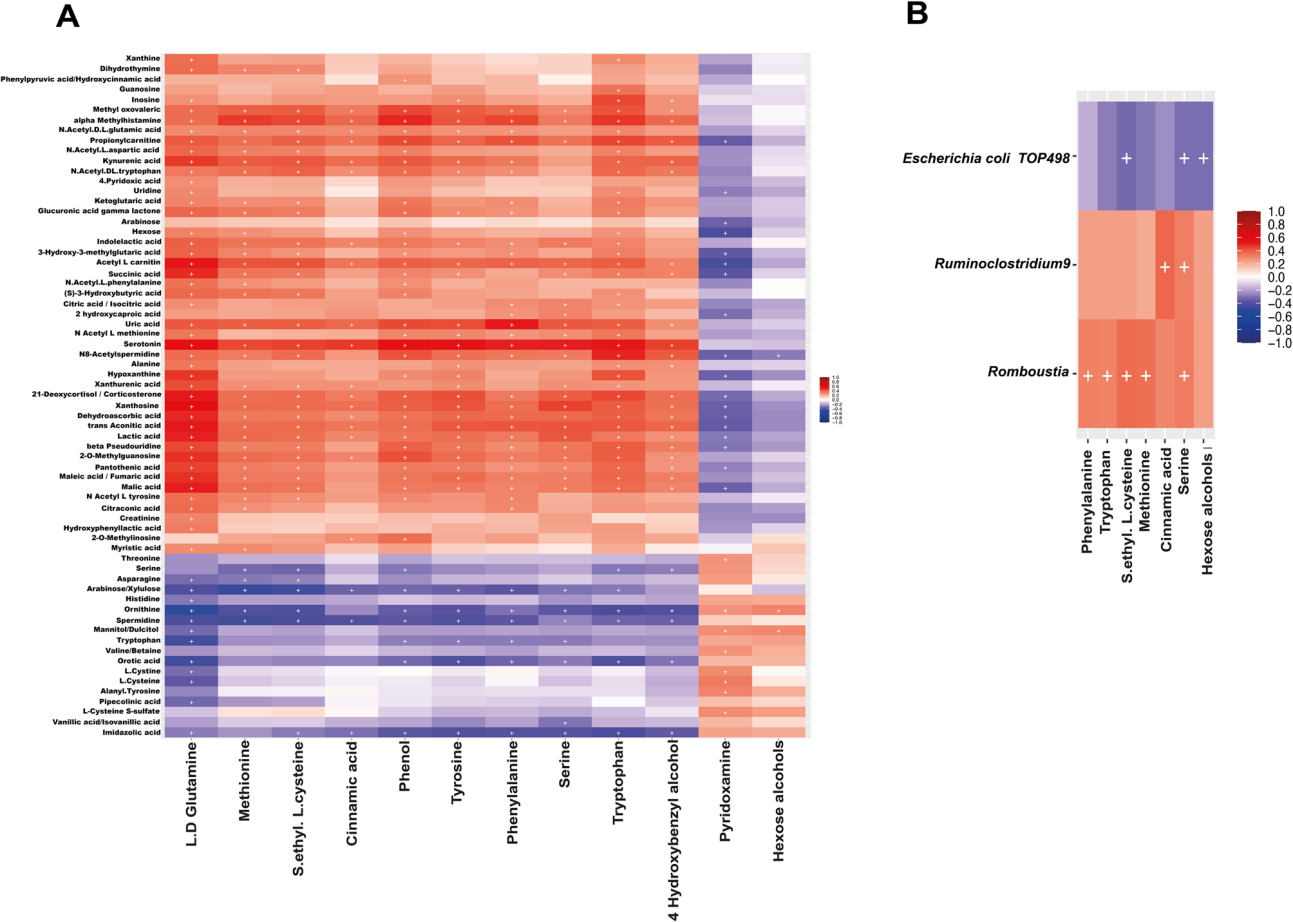
Heatmaps showing significant statistical correlations values (r) between: (**A**) serum and fecal metabolites, and (**B**) microbial taxa and fecal metabolites. Significant correlations are marked with a cross (p-adjusted value < 0.05).

To examine for correlations between the metabolites detected in serum and feces and the changes in the gut microbiota, we performed several correlation analyses of the differentially detected metabolites and the 6 bacterial taxa that varied in the gut microbiota. There was a high number of plasma and feces metabolites that were significantly associated (Fig. 5A); however, none of the associations was strong (with ρ coefficient above 0.7). The fecal metabolites glutamine and tryptophan showed the highest number of moderate associations with serum metabolites (Fig. 5A). Some of the moderate positive associations were found between the fecal metabolite glutamine and serum xanthosine, hypoxanthine and deoxyadenosine, serotonin, acetyl-carnitine, 21-deoxycortisol, kynurenic acid, lactic, malic and succinic acid and pantothenic-acid (Fig. 5A). Serum phenylalanine was positively associated with fecal serotonin, formyl-methionine, alpha-methylhistamine and propionylcarnitine. Although some of the metabolites found in serum have a microbial origin, no clear associations between differential microbial taxa and serum metabolites were found. Correlation analysis between bacterial taxa and fecal metabolites showed 9 significant correlations, of which 5 were positive correlations between the *Romboutsia* genus and the metabolites *S*-ethyl-cysteine, methionine, serine, phenylalanine and tryptophan, two positive correlations between the *Ruminiclostridium 9* genus and serine and cinnamic acid, and two negative correlations between *E. coli* TOP498 and the metabolites serine *S*-ethyl-cysteine and 4-hydroxybenzyl alcohol (Fig. 5B).

Multivariate association with linear models (MaAsLin) analysis was performed to analyze the associations between fecal and serum metabolites and significantly changed fecal microbiota taxa. Results showed that the presence of *Ruminiclostridium 9* was partly predicted by fecal phenylalanine, serum tryptophan and uric acid metabolites (positive association), and negatively associated with the presence of succinic acid and 21-deoxycortisol serum metabolites (Table 4).

**Table 4:** Regression analysis results using MaAsLin.

Bacterial taxa	Metabolite	Regression coefficient	P-adj value
*D_5__Ruminiclostridium9;D_6__uncultured Clostridia bacterium*	Fecal phenylalanine	0.3472	0.0067
	Serum tryptophan	0.4493	0.0003
	Serum uric acid	0.36942	0.0019
	Serum Succinic-acid	− 0.2486	0.0387
	Serum 21-Deoxycortisol	− 0.4142	0.0387

## Discussion

We previously showed that the performance of physical exercise in compliance with World Health Organization recommendations has a positive effect on the gut microbiota, increasing its diversity and complexity, and promoting the colonization of bacteria with potential health benefits^2,15^. To study how physical exercise modifies the gut microbiota, we designed a laboratory-based, moderate-intense exercise study to simultaneously investigate the potential changes that a single exercise bout induces in the serum and fecal metabolome and in the gut microbiota. During and immediately after exercise, there is a rapid upregulation of several metabolic pathways responsible for skeletal muscle substrate utilization, and a physiological response has been shown to occur in many organs^12,16^. Some of these changes do not seem to be completely reversed, as several of these metabolites can be considered markers of fitness status^17^. We conducted the present study with amateur athletes as it has been described that muscle signaling is more pronounced in trained people than in untrained people^18^. Our results reveal the activation of various metabolic pathways by exercise performance until volitional exhaustion. We found an increase in carbohydrate metabolites (hexoses, among others) and myo-inositol, reflective of gluconeogenic influx, which is typically induced after an initial depletion of glucose and glycogen stores^19^. Dysregulation of aminoacyl-tRNA biosynthesis reflects modifications in protein synthesis, with perturbations in proteinogenic amino acid levels. Consistent with a prior study using a maximal exercise cycling test^12^, we detected a decrease in serum tryptophan levels associated with an increase in indolelactic acid, a metabolite of the tryptophan-indole pathway^20^. Our results are also in accord with those reported after a single bout of endurance exercise^21^, with a decrease in serum tryptophan and an increase in kynurenic acid, increasing the kynurenic/tryptophan ratio. As previously reported by Lewis et al.^17^, we detected an increase in the abundance of metabolites associated with purine metabolism, including inosine, xanthine and hypoxanthine and uric acid. Of interest, hypoxanthine and xanthine accumulation in blood was also recently reported in response to a session of acute exercise^22^. The accumulation of intermediate metabolites of the tricarboxylic acid cycle, including aconitic acid, ketoglutaric acid, succinic acid, malic acid, citric acid and fumaric acid, and also lactic acid, was also evident, likely due to the activation of this pathway for ATP production, indicative of high energy demand. We also detected a slight but significant increase in hydroxybutyrate, a marker of ketone body production resulting from exhaustive exercise. Hydroxybutyrate is synthesized in the liver from fatty acids or ketogenic amino acids and can be used as an energy source by peripheral tissues when blood glucose is low^23,24^. The elevation of some acetylated amino acids and the decrease in pantothenic acid might also reflect an increased availability of acetyl-CoA by both pyruvate oxidation and beta-oxidation^17,25^. The involvement of the arginine biosynthesis pathway could be reflected by the observed decrease of spermidine and the increase of n(8)-acetylspermidine. Spermidine is a polyamine with anti-inflammatory effects, and it has been shown to enhance cell and organ function through autophagy and improve the bioavailability of arginine (not significant in the present study) required for nitric oxide biosynthesis^26^. The systemic levels of spermidine depend on oral intake, microbiota production and cellular metabolism^26^, but to our knowledge, this is the first time that its levels have been associated with exercise performance.

Exercise also induced changes in the fecal metabolome and in the gut microbiota. Metabolomic analysis revealed that the most relevant changes were in phenylalanine, tyrosine and tryptophan metabolism, reflected by an increase in tryptophan, tyrosine and phenylalanine metabolites. These essential amino acids are synthesized by gut microbiota, as humans (all animals) lack the shikimic pathway. Our results are similar to those of Zhao et al. in a half marathon running study, in which an increase of the phenylalanine, tyrosine and tryptophan biosynthesis pathway was also observed^13^. The analysis of the behavior of the microbiota regarding tryptophan synthesis during exercise is of great interest, as microbiota-contributed tryptophan in blood could counteract the decrease of tryptophan that is involved in the appearance of fatigue^27^, decreasing the effect of kynurenine—a metabolite that restricts exercise capacity^28^ and also related to depression and schizophrenia^29^. We detected kynurenine both in serum and feces, but it was not significantly modified by exercise in either compartment. While several studies have attempted to modify tryptophan levels in blood to delay central fatigue^30–32^, to the best of our knowledge no microbiota-based strategy has been proposed to increase circulating tryptophan levels. As mentioned, the microbiota plays an important role in tryptophan biosynthesis and catabolism. Because tryptophan is required for the survival of bacteria, they are able to synthesize this amino acid through a highly regulated tryptophan-biosynthesis operon, and the capacity of tryptophan synthesis is widely distributed in bacteria^33^. Bacteria also participate in tryptophan catabolism, which is directly or indirectly involved in the three major tryptophan metabolism pathways^20^. Furthermore, bacterial tryptophan metabolites influence host health, and contribute to the intestinal and systemic homeostasis in health and disease^34^, and our study suggests a strong cross-talk between microbiota and systemic tryptophan metabolism. Fecal tryptophan levels correlated negatively with the presence of *E. coli* TOP498 and positively with the presence of the *Romboutsia* genus. Also, we found changes in the abundance of *E. coli* and several bacterial taxa (*Romboutsia*, *Ruminococcocaceae UCG-005*, *Blautia*, *Ruminiclostridium 9* and *Clostridium phoceensis*) belonging to the Clostridia class, which possess tryptophan synthesis machinery^33^.

We detected an increase in fecal ammonia after the exercise bout, which could be related to the metabolism of urea and glutamine that can occur during exercise, as both metabolites are physiological markers of exercise performance^35^. The main sources of ammonia in the gastrointestinal tract are from urea degradation and glutamine metabolism, and from dietary proteins^36^. Urea produced during exercise can permeate the intestinal tract and serve as a fuel for bacteria, enabling the growth of urease-containing bacteria^36^. The transfer of metabolites produced during exercise from the blood to the gastrointestinal system could be a mechanism by which exercise induces changes in the microbiota, as some of the bacteria detected in this study including *Romboutsia, Ruminococcus* and *Clostridium* exhibit urease activity^37,38^. In fact, a recent study demonstrated that the lactate produced during exercise can increase the abundance of the *Veillonella* genus in gut microbiota^39^.

The present study has some limitations that should be considered. The time of collection of the stool samples was not concurrent with the collection of serum samples. The timing for serum collection between T1 and T2 was clearly established; however, the collection of the stool samples was not as precise, although we limited the time to 4 h to avoid any other external influence. This could account for the lack of correlation between differential microbial taxa and serum metabolites.

In conclusion, we provide evidence that a single bout of acute physical exercise in amateur athletes triggers changes in serum and fecal metabolism and also in gut microbiota. The changes we have seen in the microbiota are subtle, as they have only been produced by an exercise bout. Despite being highly trained individuals the exercise they performed was very intense for them, reaching exhaustion, which can clearly be seen in the metabolomics approach. Further research is needed to better understand the interaction between the human body and the trillions of bacteria that inhabit it, as well as the changes induced by exercise on the gut microbiota and associated mechanisms. Exercise frequency, intensity, performing time, type of exercise, exercise volume and progression are all factors that influence physiological responses and exercise adaptations, and will need to be considered in future studies investigating the beneficial effect of exercise on the gut microbiota.

## Materials and methods

### Study design

The present study is a single-arm trial. Participants were recruited from different cross-country athletes' teams in Madrid, Spain. From the 68 participants screened, 40 male endurance cross-country athletes met the following inclusion criteria: 18–50 years of age, with a high physical condition (oxygen uptake VO_2_ ≥ 55 mL/kg/min), and body mass index 18–25 kg/m^2^ and treadmill experience. Exclusion criteria were antibiotics intake during 3 months prior to the study, smoking, nutritional or ergogenic supplements, prebiotics, probiotics, be vegetarian or vegan, chronic medication, gastrointestinal surgery, or any diagnosed disease. Participants were also excluded if they had any medical condition that could be exacerbated by exercise. The Ethics Committee for Clinical Research of the Community of Madrid Spain approved the study (Ref: 07/694487.9/17), and all procedures were in accordance with the 1964 Helsinki Declaration and its later amendments. Written informed consent was obtained from all the volunteers. The study was registered in Clinicaltrials.gov with the accession number NCT04244149.

### Dietary habits

Dietary pattern characterization of the participants was carried out using a validated food frequency questionnaire with 93 food items^40^ and three 24-h dietary recalls (two weekdays and one weekend day). Data from the questionnaires were analyzed using Dietsource software 3.0 (Novartis, Barcelona, Spain) to obtain the total energy ingested (in kcals) of proteins, fat, carbohydrates and fiber.

### Exercise protocol

Body weight and height were measured with a scale and a height meter (Asimed T2, Barcelona, Spain). BMI was calculated by dividing the weight by the square of the height. Participants refrained from any physical activity 24 h before the day of the physical test. All participants performed a standardized 10-min warm-up of continuous running on a treadmill (H/P/Cosmos Venus, Nussdorf-Traunstein, Germany) at 60% of their maximum heart rate. After the warm-up, they ran with a slope of 1% at a speed of 10 km/h, with increments of 0.3 km/h every 30 s until volitional exhaustion. Participants were verbally encouraged to give their maximal effort, particularly towards the end of the test. Oxygen consumption values were monitored during the test and the following variables were determined: VO_2_, pulmonary ventilation, ventilatory equivalents for oxygen and carbon dioxide, and end-tidal partial pressure of oxygen and carbon dioxide. These variables were used to calculate absolute maximal oxygen consumption (VO_2maxABS_), relative maximum oxygen consumption (VO_2maxREL_), maximal aerobic speed, and first and second ventilatory thresholds. After the exercise test, the participants performed a 1-km run on an athletics track at maximum speed. The time needed to complete the run was also measured.

### Serum and stool sample collection

Venous blood samples were collected in vacutainer tubes immediately before and within 15 min of finishing the exercise test (T1 and T2, respectively). Serum was obtained after clotting and centrifugation at 760×*g* for 10 min at 4 °C; aliquots were immediately frozen and stored at − 80 °C. Stool samples were collected just before and after the exercise session. Participants were provided with a Fe–col stool collection device, an insulated bag and ice blocks to preserve the samples until they were delivered to the laboratory. The first stool sample was collected early in the morning, before physical exercise. The second sample was collected within four hours post-exercise to avoid diet interference. Exercise promotes bowel transit and normally, participants feel the need to defecate soon after intense exercise. Those participants who did not produce a stool sample within four hours after exercise were excluded from the study. On arrival at the laboratory, the samples were maintained at − 80 °C until processing. Stool samples for metabolomics analysis were lyophilized using the TFD5503 Bench-Top freezedryer (ilShin Biobase, Ede, Netherlands) and stored at − 80 °C.

### Chemicals and reagents

All analytical grade reference compounds were from the Sigma Chemical Co. (Saint Quentin Fallavier, France). The standard mixtures used for the external calibration of the mass spectrometry (MS) instrument (Calmix-positive, for the positive ion mode, consisting of caffeine, l-methionyl-arginyl-phenylalanyl-alanine acetate; and Ultramark 1621, and Calmix-negative, for the negative ion mode, consisting of the same mixture plus sodium dodecyl sulfate and sodium taurocholate) were from Thermo Fisher Scientific (Courtaboeuf, France). Acetonitrile was from SDS (Peypin, France), formic acid from Merck (Briare-le-Canal, France), methanol from VWR Chemicals (Fontenay-sous-Bois, France) and deionized water from Biosolve chemicals (Dieuse, France).

### Metabolite extraction from stool and serum and analytical measurements

Approximately 2 × 10 mg of lyophilized stool were weighed precisely in two distinct Precellys tubes (ref CK14-2 mL, Bertin Technologies, Montigny-le-Bretonneux, France), resuspended in 150 µL of pure water and thoroughly vortexed. Then, 600 µL of methanol were added. After vortexing, samples were lysed in a Precellys Device (Bertin Technologies) for 3 × 30 s at 6500 rpm and 4 °C, and were then left on ice for 90 min to facilitate complete protein precipitation.

Two 50-µL samples were withdrawn from each serum sample. Each sample was mixed with 200 µL of methanol and incubated on ice for 90 min to achieve protein precipitation.

After centrifugation at 20,000*g* for 15 min at 4 °C, 200 µL of the supernatant were withdrawn and evaporated to dryness under a nitrogen stream at 30 °C using a Turbovap instrument (Caliper Life Science Inc., Roissy, France). The resulting dried extracts were stored at − 80 °C until analysis. Dried aliquots were resuspended in either 100 µL of water/acetonitrile (95:5, v/v) with 0.1% formic acid for C18 analysis or 100 µL of a mixture of 10 mM ammonium carbonate buffer (pH 10.5) and acetonitrile (40:60) for ZIC-pHILIC analysis (see below). The tubes were then vortexed, incubated in an ultrasonic bath for 5 min, and centrifuged for a further 10 min. A volume of 95 μL of the supernatant was transferred to 0.2 mL-vials and mixed with 5 µL of a mixture of internal standards (^13^C-glucose, ^15^N-aspartate, ethylmalonic acid, amiloride, prednisone, metformin, atropine sulfate, colchicine, imipramine) in order to check for consistency of analytical results in terms of signal and retention time stability throughout the experiment. Quality control samples were obtained by pooling aliquots of each sample and these were injected every 5–10 samples throughout the analysis for further data normalization/standardization.

Untargeted metabolomics experiments were performed by liquid chromatography coupled to high-resolution mass spectrometry (LC-HRMS) using a combination of two complementary chromatographic methods^41,42^, consisting of reversed-phase chromatography (C18 chromatographic column) and hydrophilic interaction chromatography (HILIC) for the analysis of hydrophobic and polar metabolites, respectively. LC-HRMS experiments were conducted on an Ultimate 3000 chromatographic system (Thermo Fisher Scientific, Courtaboeuf, France) coupled to an Exactive mass spectrometer from Thermo Fisher Scientific fitted with an electrospray ionization source and operating in the positive and negative ion modes for C18 and HILIC separations, respectively (designated as C18(+) and HILIC(−), respectively). Metabolite separations were performed using a Hypersil GOLD C18 1.9 µm, 2.1 × 150 mm column maintained at 30 °C (Thermo Fisher Scientific) or a Sequant ZIC-pHILIC 5 µm, 2.1 × 150 mm column at 15 °C (Merck, Darmstadt, Germany), operated under gradient elution conditions as described^41^.

### Metabolomic data processing, metabolite annotation and metabolic pathway analysis

The raw LC-HRMS data were first converted into mzXML files using MSConvert (ProteoWizard), and further data processing (e.g., normalization, scaling, filtering) and statistical treatments were performed using the Workflow4Metabolomics (W4M) platform^43^. Metabolite annotation was first realized using our spectral database according to accurately measured masses and chromatographic retention times^41^. Our chemical database initially included ~ 1000 metabolites mainly of human origin, but was then broadened by the addition of commercially available bacterial metabolites and other molecules described in the literature^44,45^. Confirmation of metabolite annotation was then accomplished by running additional LC–MS/MS experiments using a Dionex Ultimate chromatographic system combined with a Q-Exactive mass spectrometer (Thermo Fisher Scientific) under non-resonant collision-induced dissociation conditions using higher-energy C-trap dissociation. Obtained MS/MS spectra were both manually and automatically matched using MS-DIAL software^46^ to the spectra included in our in-house spectral database, as described^47^. To be identified, ions had to match at least 2 orthogonal criteria (accurately measured mass, isotopic pattern, MS/MS spectrum and retention time) to those of an authentic chemical standard analyzed under the same analytical conditions, as proposed by the Metabolomics Standards Initiative^48^. The identified metabolites were imported into the free online web-based platform MetaboAnalyst 4.0^49^ for metabolic pathway enrichment. Thus, the annotated *m/z* features with a Wilcoxon p-value < 0.005 (BH-critical value) were imported as KEGG numbers using the appropriate *Homo sapiens* pathway library. The interpretation of the results was performed after considering data with a p-value < 0.05 and an impact value > 0.1.

### pH and ammonia determination in stool samples

The pH was measured using a basic pH meter (Crisom, Hach Lange, Barcelona, Spain) according to the method described by Dai and Karring^50^. Ammonia content was determined using a high-performance ammonia-selective ion electrode (Orion, Thermofisher Scientific, Waltham, MA). Stool (100 mg) was dissolved in 5 mL of MilliQ water; the mixture was vortexed for 2 min and then sonicated (Ultrasons, Selecta, Barcelona, Spain) for 10 min. A volume of 100 µL 1 M NaOH was added to the sample and ammonia was immediately measured. A standard curve was made from serial dilutions of ammonium chloride from 0.1 M, according to the manufacturer's instructions.

### Bacterial DNA extraction and bioinformatics

Bacterial DNA was extracted using the E.Z.N.A. Kit (Omega-Biotek, Norcross, GA) and a bead homogenizer (Bullet Blender Storm, Next Advance, Averill Park, NY). A DNA fragment comprising the V3 and V4 hypervariable regions of the 16 s rRNA gene was amplified for sequencing analysis^6^. Amplicons were sequenced on the MiSeq Illumina platform (Illumina, San Diego, CA). Sequence results were analyzed using Quantitative Insights into Microbial Ecology (QIIME2) v2019.7^51^ and were processed with DADA2 for quality control^52^. The classify-sklearn method was used for taxonomy assignment^53^ with an in-house customized classifier based on the SILVA reference database^54,55^. To construct the customized reference database, we extracted the sequences according to our primers (forward primer sequence: CCTACGGGNGGCWGCAG, reverse primer sequence: GACTACHVGGGTATCTAATCC) from the SILVA 132 database clustered at 99% identity^56^. We trained the classifier using our tailored reference reads and SILVA 7-levels for reference taxonomy, including the species probability (weights) likely to be observed for human stool (downloaded from https://github.com/BenKaehler/readytowear)^56,57^.

### Statistical analysis

For metabolomics data, multivariate analyses were used to identify molecular features that discriminate metabolite differences in athletes before and after a session of acute exercise. PCA and PLS-DA were performed using the W4M platform and were used to identify features with discriminative power and to maximize variation between the two groups (before and after the test). Also, permutation tests (200 cycles) were conducted to assess the robustness of the PLS-DA model when using a small sample size^58^. The significance of the discriminant metabolites from the two groups was defined by a statistically significant threshold of VIP, which was derived from the PLS-DA model. A value of VIP > 1.0 was considered sufficient for group discrimination^59^. The univariate data analyses included a Wilcoxon signed-rank test, corrected for multiple testing by the Benjamini–Hochberg procedure, to assess the statistical significance of each compound. The generation of the clustered heatmap was performed using the Canberra distance metric and normalizing data, adding a pseudocount of 1 before log10 conversion. To detect changes in microbiota, samples in T1 and T2 were compared using the q2-longitudinal method^60^. First, a feature volatility analysis was performed to explore the data. This type of analysis uses a supervised learning regressor to predict a continuous variable (time in this case) as a function of feature composition (bacterial taxa). Based on volatility analysis, some bacterial taxa were selected for subsequent analyses according to their importance and cumulative average change. Selected taxa were analyzed using a linear mixed-effects model to detect significant bacterial taxa. Associations between different variables were studied with Spearman´s correlation and multivariate association by linear models (MaAsLin), an additive general linear model to find associations between metadata and bacterial abundance^61^. The cumulative sum scaling normalization was used. All p-values were corrected for multiple testing using the Benjamini–Hochberg false discovery rate.

## Supplementary Information

Supplementary Information 1.

Supplementary Information 2.

## Data Availability

The 16S rRNA dataset generated during the present study has been submitted to the NCBI Biosample database, https://submit.ncbi.nlm.nih.gov/subs/sra/SUB7780980/overview and will be available upon publication.
