# Supplementary material for: The effect of acute moderate-intensity exercise on the serum and fecal metabolomes and the gut microbiota of cross-country endurance athletes
Source: Sci Rep. 2021 Feb 11;11:3558. doi: 10.1038/s41598-021-82947-1 (PMC7878499; doi:10.1038/s41598-021-82947-1)
Supplement: Supplementary file 2 — Supplementary Information 2. [file 41598_2021_82947_MOESM2_ESM.pdf]

The effect of acute moderate-intensity exercise on the serum and fecal metabolomes and the gut microbiota of cross-country endurance athletes

Mariangela Tabone[1†], Carlo Bressa[1†], Jose Angel García-Merino[1], Diego Moreno-Pérez[2], Emeline Chu Van[3], Florence A. Castelli[3] François Fenaille[3*], Mar Larrosa[1*]

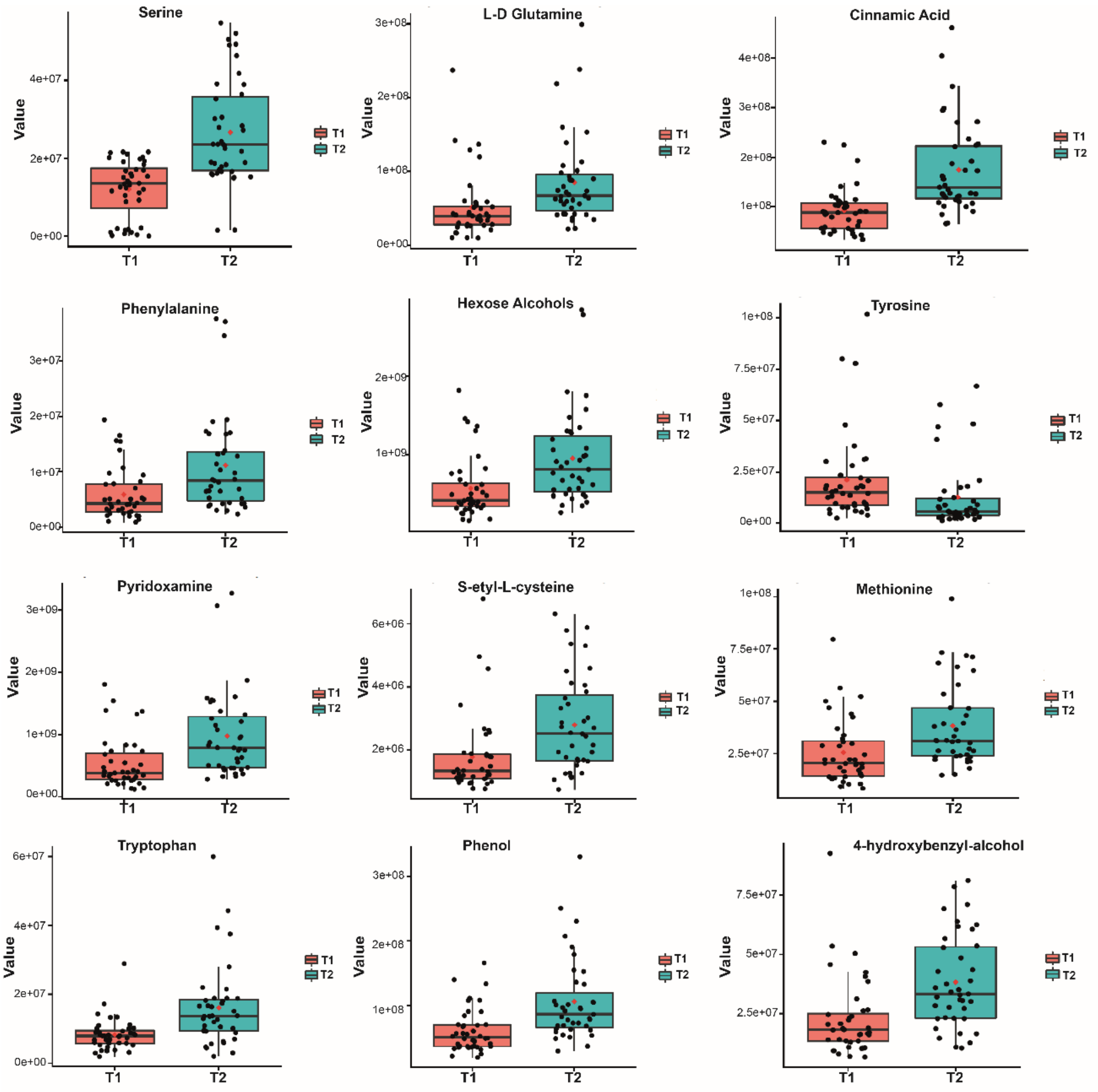

Figure S1: Fecal metabolites whose peak intensity is significantly different between T1 and T2 (p-adjusted < 0.05).

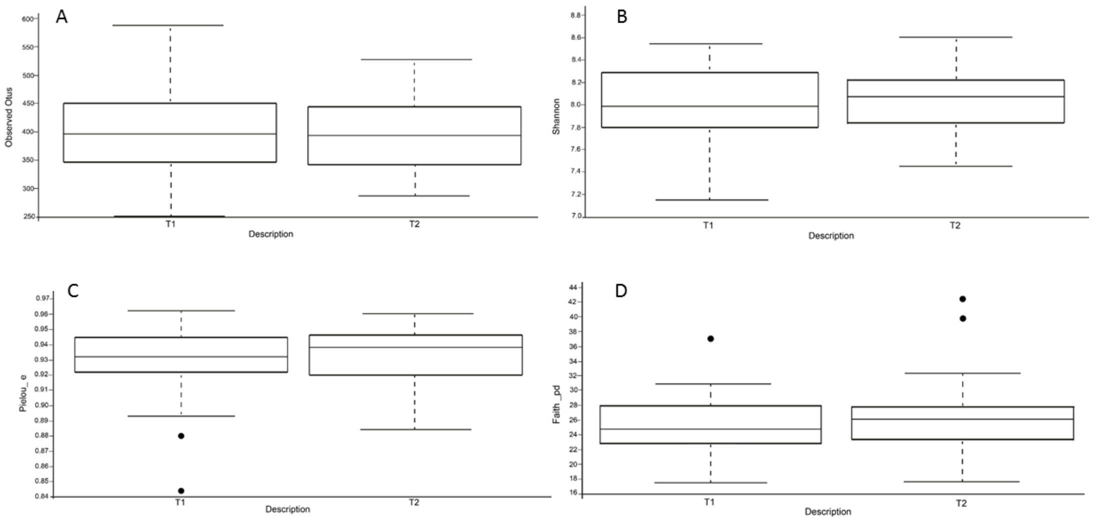

Figure S2: Box plots showing alpha diversity, A) Observed OTUs, B) Shannon Index, C) Pielou's evenness index and D) Faith's Phylogenetic Diversity index in T1 and T2.
